# Providing inclusive, person‐centred care for LGBT+ older adults: A discussion on health and social care design and delivery

**DOI:** 10.1111/jonm.13178

**Published:** 2020-12-01

**Authors:** Lorna Roe, Miriam Galvin

**Affiliations:** ^1^ Global Brain Health Institute Trinity College Dublin, the University of Dublin Ireland; ^2^ Centre for Health Policy and Management Trinity College Dublin, the University of Dublin Ireland; ^3^ The Irish Longitudinal Study on Ageing Trinity College Dublin, the University of Dublin Ireland; ^4^ Academic Unit of Neurology, School of Medicine Trinity College Dublin, the University of Dublin Ireland

**Keywords:** cultural competency, health policy, health services accessibility, healthy ageing, sexual and gender minorities

## Abstract

**Aim:**

To examine how health system design and delivery can fail to support the needs of LGBT+ older adults.

**Background:**

LGBT+ older adults face barriers in access to care, impacting their ability to receive person‐centred care in old age, which is central to the prevention and management of frailty, disability and disease.

**Evaluation:**

Using a conceptual framework of access to care, this commentary illustrates issues LGBT+ older adults may face in accessing health and social care services in Ireland, and provides examples of how access may be improved from the published international literature.

**Key issue(s):**

Health policies, service design and delivery all impact on the ability of the health system to meet the needs of LGBT+ older adults across all levels and types of care.

**Conclusion:**

Heteronormativity and discrimination must be addressed across the whole health system to achieve the health policy goal of supporting all older adults to enjoy health and well‐being.

**Implications for nursing management:**

We suggest nursing professionals use a systems perspective to address the multilevel issues relating to care for LGBT+ older adults. Researchers in gerontological nursing should include the experiences and outcomes of service utilization for LGBT+ older adults in their research agenda.

## 1 INTRODUCTION

Those who identify as lesbian, gay, bisexual and transgender/transsexual (LGBT+) are described as having an invisible identity, and LGBT+ older adults as being ‘doubly invisible’ (Higgins et al., 2011), particularly within health research (Institute of Medicine, 2011). This commentary uses examples from the Irish health system to illustrate how older LGBT+ people may encounter barriers in accessing care, due to the design of the health care system and the model of service delivery. Issues addressed are viewed using a conceptual framework of access to health care, and we discuss solutions drawing on examples from the Irish and international literature, and the contribution which nursing professionals working in clinical, research, management or policy roles can make.

## 2 BACKGROUND

Research addressing ageing and the LGBT+ community has increased in recent years (Fredriksen Goldsen et al., 2019; Fredriksen‐Goldsen & Muraco, 2010). Lifetime exposures to stress resulting from societal or familial rejection, discrimination and internalized stigma among other factors are associated with poor later life health outcomes (Fredriksen Goldsen et al., 2019). There is evidence that LGBT+ older adults face health inequities in comparison with non‐ LGBT+ older adults (Fredriksen Goldsen et al., 2019). Some LGBT+ older adults are found to have psychosocial health benefits such as resilience (Higgins et al., 2011), and strong social networks (Roe et al., 2020). However, others report ageism and alienation from within their own community (Fredriksen Goldsen et al., 2019; Higgins et al., 2011; Hoy‐Ellis et al., 2016). A large proportion of LGBT+ older adults live alone (Higgins et al., 2011), which can increase the risk of poverty and the need for paid homecare in old age (Central Statistics Office, 2015; Murphy et al., 2014). LGBT+ older adults (notably gay men) are more likely to have experienced the death of a partner, disenfranchised grief and survivor guilt (Fredriksen Goldsen et al., 2019).

There is evidence to show the support and health needs of LGBT+ older adults are not always adequately addressed within health systems (Donaldson & Vacha‐Haase, 2016; Fredriksen Goldsen et al., 2019; Roe et al., 2020). Without training in cultural competency, care providers can be unprepared when working with LGBT+ older people (Holman et al., 2020). There is a call for more research on health care services' capacity to deliver LGBT+ affirmative health care and associated education and training needs (Westwood et al., 2020). Nursing professionals can play a vital role in addressing these issues; they are frequently patient‐facing, the first point of contact for service users, and are integrated across health systems (American Nurses Association NA, 2015).

## 3 THE IRISH HEALTH SYSTEM AS A CASE EXAMPLE

In Ireland, as elsewhere, there is an explicit policy to support older adults to enjoy physical and mental health and well‐being to their full potential (Department of Health, 2013). Health systems play an important role by delivering care to prevent or manage conditions commonly seen in old age such as frailty, disability and disease. Up to seven per cent of the population aged ≥55 years in Ireland identifies as LGBT+ (Higgins et al., 2011). The number of LGBT+ older adults is expected to increase with population ageing and higher rates of disclosure. LGBT+ older adults report receiving poor quality services, and recommended services are more overtly inclusive of LGBT+ older people (Fredriksen Goldsen et al., 2019; Higgins et al., 2011).

## 4 ACCESS TO CARE: A CONCEPTUAL FRAMEWORK

**Figure 1:**
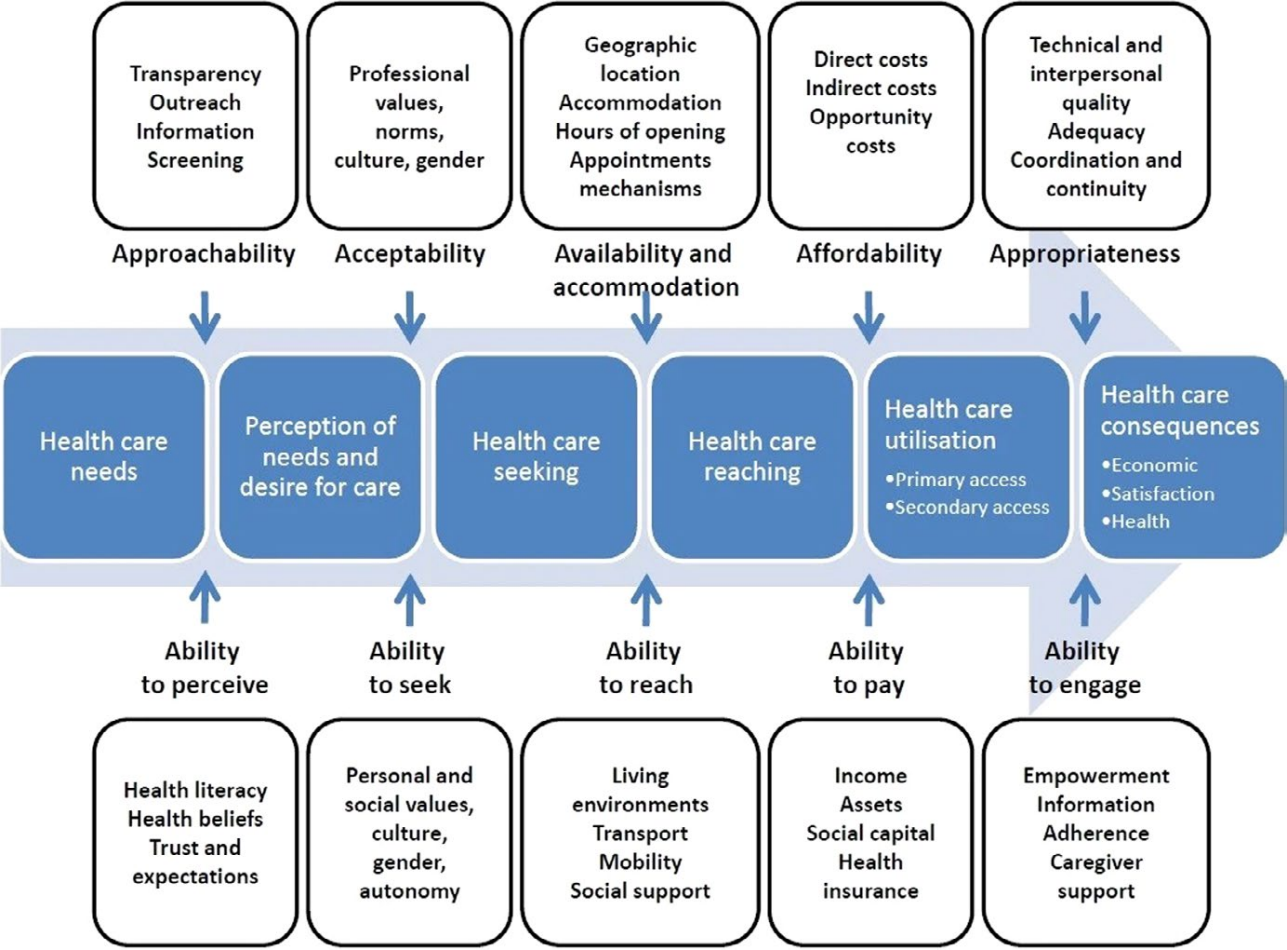
A conceptual framework of access to health care (Levesque et al., 2013). Figure reproduced from Levesque et al. (2013) under the terms of the Creative Commons Attribution 2.0 Generic Licence (CC BY 2.0), which permits unrestricted use, distribution and reproduction in any medium, provided the original work is properly cited [Colour figure can be viewed at wileyonlinelibrary.com]

Access to health care is defined as the opportunity to reach appropriate health care services in situations of perceived need (Levesque et al., 2013). We examine how systems may/not meet the needs of LGBT+ older adults by exploring the characteristics of health systems, organisations and providers (see Figure 1).

### 4.1 Approachability

*Approachability* means that people can identify that service(s) exist and can be obtained to address their health needs. For some LGBT+ older adults, prior experiences of discrimination or stigma within health services mean they may not view these services as inclusive spaces where they will be respected, and where their needs will be met (Fredriksen‐Goldsen et al., 2018; Higgins et al., 2011; Roe et al., 2020). Services specifically addressing the needs of LGBT+ older adults may be a solution such as LGBT+ dementia care services (Fredriksen Goldsen et al., 2019; Opening Doors London, 2020), LGBT+ senior housing (Mayor's Office of Housing and Community Development, 2020) and non‐governmental organisations, which are set up to advocate for the needs of LGBT+ older adults and provide support services (SAGE, 2020). However, these services may be hard to reach, or may not exist, in countries where it is not acceptable or safe to conduct research on, or to advocate for LGBT+ issues (Fredriksen Goldsen & de Vries, 2019). Nurses in management roles can drive innovation and influence nursing policy to address the needs of LGBT+ older adults (American Nurses Association NA, 2015).

### 4.2 Acceptability

*Acceptability* means that services are culturally or socially acceptable to people. In Ireland, only one third of LGBT+ older adults believed that health care professionals had sufficient knowledge about LGBT issues, and less than half felt respected as an LGBT+ person by health care providers (Higgins et al., 2011). Internationally, staff may wish to provide affirming, high‐quality care to LGBT+ older adults, but have little knowledge of LGBT‐specific health concerns, or to signpost to agencies which provide LGBT+ specific services (Donaldson et al. 2016). In the management of complex needs, such as frailty, older adults become users of many different kinds of services within the health system (Roe et al., 2017). Thus, LGBT+‐specific services could address some, but not all, of the support and health needs of LGBT+ older adults. The health system must be able to provide inclusive, person‐centred care at all points of contact. This does not mean that clinics must create new protocols to accommodate each diverse user or that all staff need to be experts on LGBT+ health. One solution is to change the way in which health and well‐being is viewed, by moving away from a solely biomedical model towards a biopsychosocial model that assesses health and well‐being holistically, in the context of a person's lived experience (Hewa & Hetherington, 1995). Organisations can change existing services and provide care that better addresses the needs of diverse end users: examples include making ‘positive spaces’ for LGBT+ older adults within organisations; developing service materials (e.g., posters), which explicitly welcome LGBT+ people; creating LGBT+ caregiver support resources; connecting mainstream services and LGBT+ organisations; and training staff to have the language and skills to engage with service users from the LGBT+ community (Fredriksen‐Goldsen et al., 2018; LGBT Ireland, 2019; MacDonnell & Daley, 2015). Nursing professionals work in different settings and disciplines within health services and are tasked with providing culturally competent services. Thus, nursing professionals need to be central to efforts to develop skills to deliver affirmative care to LGBT+ older adults.

### 4.3 Availability or accommodation

*Availability or accommodation* means that health services can be reached physically and in a timely manner. Policies and operational service rules may have a heteronormative bias (Fredriksen Goldsen et al., 2019). Take for example the case of homecare in Ireland, where 90% of carers for older adults are informal or unpaid carers, 70% of whom are women (most commonly a female spouse) (Kamiya et al., 2012). The reliance on female‐led, familial informal care in Ireland can be understood in its historical context. The Irish State has historically provided a safety‐net level of homecare as opposed to universal provision (Timonen & Doyle, 2008) and highlights women's role in the family within the Irish constitution. This socially conservative approach is said to have been influenced by Catholic social teaching, which advocated for the primacy of the family and community in the provision of social services, with state involvement as a last resort (i.e., the principle of subsidiarity) (Evans, 2013). These factors contribute to today's system, in which the state only intervenes when the family's capacity to service its members is exhausted (Adshead & Miller, 2003). The reliance on female‐led, familial homecare is heavily based on a heteronormative assumption, which has created a system that may not readily accommodate the needs of LGBT+ older adults who may not have this kind of familial support available. Nursing professionals working on policy development can raise awareness of these issues among politicians, other policymakers and stakeholders and address the ways in which policies fail to address the needs of LGBT+ older adults.

### 4.4 Affordability

*Affordability* of care relates to the financial cost of care that facilitates or impedes access to care. In Ireland, older adults living alone are more likely to use state formal homecare services (Murphy et al., 2014); however, the level of state support is capped, requiring the person to supplement this for an adequate level of care. However, the cost of private homecare may be beyond the means of many households, particularly single occupancy, female‐led households. The situation is exacerbated by resource allocation within the public long‐term care system. Older adults have a statutory right to nursing home care, but the same rights do not extend to homecare, which is delivered as an administrative scheme where budgets can vary by year. The system of resource allocation and poor availability of informal care can place LGBT+ older adults at risk of early admission to nursing home care, which has been found to be the least preferred option for older age accommodation for LGBT+ older adults (Higgins et al., 2011).

### 4.5 Appropriateness

*Appropriateness* refers to the fit between services and the needs of users in terms of timeliness of care, needs assessment, care planning and the interpersonal quality of the services provided. Heteronormativity within health systems can be seen in service assessment forms, which fail to collect information on sexual orientation and gender identity. Knowing this information could influence care plans and prompt staff to involve ‘chosen family’; use preferred pronouns; include same‐sex partners; assist appropriate room placement in nursing home care; and ensure LGBT+ older adults are cared for in a way they would have chosen at the end of life (Cartwright et al., 2012; Daley et al., 2016; de Vries et al., 2020; Donaldson & Vacha‐Haase, 2016; Fredriksen Goldsen et al., 2019; MacDonnell & Daley, 2015; Roe et al., 2020). This information would also help staff clinically, for example managing age regression delusions in dementia, or ensuring transgender women are given proper screening for prostate cancer (Donaldson et al. 2016). The inclusion of details on gender identity and sexual orientation in assessment forms would make older LGBT+ people who are not connected to traditional heteronormative systems of support ‘visible’ to care providers (De Vries et al., 2020).

In a home setting, it is more difficult to monitor discrimination and abuses by service providers. This is troubling as the home was often a sanctuary for LGBT+ people, who report hiding their identity when health care staff visit their home, for example removing photographs that would disclosure their identity (Daley et al., 2016; Roe et al., 2020). In Ireland, safeguarding standards do not explicitly refer to risks faced by LGBT+ older adults, and without a legal basis, there remains no official inspection system in place to regulate standards (Health Information and Quality Authority, 2019). Nurses working in the community are well placed to identify and report discriminatory behaviour towards LGBT+ older adults, and to advocate for their needs.

## 5 LIMITATIONS OF THE COMMENTARY

This commentary draws on studies about the support needs of LGBT+ older adults in the published literature. Studies not in English‐language publications have not been addressed. We illustrate ways in which LGBT+ older adults experience services; however, we do not suggest these issues affect *all* LGBT+ older adults or are present in every health system. Although we provide examples of good practice within the literature, we suggest efforts to improve services locally are undertaken with the explicit involvement of local LGBT+ older adults.

## 6 CONCLUSION

This commentary reveals how policies, operational service rules, models of resource allocation, service assessment forms, knowledge and practice, and safeguarding regulations may not be designed or delivered in such a way as to meet the needs of LGBT+ older adults. These issues were identified across different levels of care (e.g., primary, secondary, tertiary and long‐term care) and types of care (e.g., geriatric care, cancer care, dementia end of life care). We conclude that addressing heteronormativity and discrimination is needed across the whole system of care, to achieve the policy goal of supporting *all* older adults to enjoy physical and mental health and well‐being to their full potential.

## 7 IMPLICATIONS FOR NURSING MANAGEMENT

Nursing professionals are well placed to address these issues given their scope of practice across the whole system of care, their involvement in policy making and commitment to delivering culturally competent care. We suggest nursing management who are charged with mentoring, training staff and service improvement recognize the importance of taking a systems perspective to improve access and care services for LGBT+ older adults. Researchers in gerontological nursing should add the experiences of service utilization for LGBT+ older adults service users to their research agenda.

## ETHICAL APPROVAL

This commentary is based on existing published literature and did not require ethical approval.
